# ANLN Enhances Triple-Negative Breast Cancer Stemness Through TWIST1 and BMP2 and Promotes its Spheroid Growth

**DOI:** 10.3389/fmolb.2021.700973

**Published:** 2021-07-01

**Authors:** Alishba Maryam, Y. Rebecca Chin

**Affiliations:** ^1^ Department of Biomedical Sciences, City University of Hong Kong, Kowloon, Hong Kong; ^2^ Key Laboratory of Biochip Technology, Biotech and Health Centre, Shenzhen Research Institute, City University of Hong Kong, Shenzhen, China

**Keywords:** Anilin, breast cancer, cancer stem cells, 3D culture, CRISPR/Cas9

## Abstract

ANLN is frequently upregulated in triple-negative breast cancer (TNBC) and its high expression in tumors are significantly associated with poor survival and recurrence, thereby it has been proposed to function as a prognostic marker for breast cancer. However, the specific function and molecular mechanisms by which ANLN promotes TNBC tumorigenesis remain elusive. Using multiomic profiling, we recently uncovered ANLN as a TNBC-specific gene driven by super-enhancer. Here, by Crispr/Cas9 editing, we showed that knockout of ANLN inhibits spheroid growth of TNBC. Interestingly, its effect on cell proliferation in 2D cultures is minimal. ANLN depletion inhibits mammosphere formation and clonogenicity potently, suggesting its important function in regulating cancer stem cells (CSCs). We screened a panel of stem cell-related genes and uncovered several CSC genes regulated by ANLN. We further identify TWIST1 and BMP2 as essential genes that mediate ANLN’s function in stemness but not spheroid growth. These findings may contribute to search for effective targeted therapies to treat TNBC.

## Introduction

Breast cancer is the most prevalent cancer in women worldwide, with an estimated 2 million new cases diagnosed in 2020 (Sung et al., 2021). Conventionally, breast cancer is classified into three major subtypes: luminal, HER2 overexpressed, and basal-like. Approximately 70% of basal-like tumors are triple-negative breast cancer (TNBC; ER^−^/PR^−^/HER2^-^) (Cancer Genome Atlas, 2012), which is more aggressive, enriched in cancer stem cells (CSCs) and prone to metastasis. Chemotherapy, usually with high toxicity, remains the major treatment option for TNBC, thus there is an urgent clinical need to identify novel therapeutic targets for this aggressive subtype. Over the past years, genomic and transcriptomic studies have revealed genetic features of different subtypes of breast cancer. However, epigenetic mechanisms in regulating oncogene expression in breast cancer subtypes remain poorly understood.

Enhancers are cis-regulatory regions that promote transcription of a target gene from a distance (Zhang et al., 2016). Super-enhancers contain clusters of enhancers that were originally shown to define tissue specificity (Herranz et al., 2014). They are characterized by high level of the histone modifications such as H3K27ac and coactivator binding. Increasing evidence has demonstrated the critical role of super-enhancers in promoting cancer development by driving expression of oncogenes (Hnisz et al., 2013). For example, MYC-associated super-enhancer was shown to be focally amplified in multiple epithelial cancers to drive MYC expression in tumors (Zhang et al., 2016). In gastric cancer, super-enhancer landscape was reprogramed during tumorigenesis, underpinning the dysregulation of cancer-related genes (Ooi et al., 2016). The enrichment of super-enhancers in different subtypes of breast cancer, however, has not been determined. To address heterogeneity of super-enhancers and their functional role in breast cancer, we recently reported a multiomic study employing ChIPseq profiling, gene expression data and network-based analyses to uncover TNBC-specific super-enhancer landscape (Huang et al., 2021). Using Crispr/Cas9 editing, we further demonstrated the functional significance of super-enhancer in driving the expression of key oncogenes FOXC1 and MET in TNBC. Harnessing the power of exploring epigenomic features in TNBC, a number of novel super-enhancer-regulated TNBC-specific genes were emerged from the study, including Anilin (ANLN).

ANLN has been demonstrated to play a key role in cytokinesis. During interphase of the cell cycle, ANLN is resided in the nucleus. Whereas during telophase, ANLN accumulates in the cytoplasm, forming a contractile ring and cleavage furrow by interacting with various proteins including myosin, F-actin, RhoA and septin (Oegema et al., 2000; Piekny and Maddox, 2010). Upregulation of ANLN is observed in various cancers including lung, pancreatic, ovarian, colorectal, hepatic and breast cancer (Magnusson et al., 2016). Studies have demonstrated that ANLN promotes tumor cell proliferation by regulating cell cycle progression. For example, in ANLN-depleted non-small lung cancer and breast cancer lines, polynucleated cells were observed and cell proliferation was inhibited (Suzuki et al., 2005; Zhou et al., 2015; Magnusson et al., 2016). In addition, breast cancer patients with high expression of ANLN showed significantly poorer overall survival (Magnusson et al., 2016), recurrence as well as higher expression of proliferation genes (all 17 tested in (Thapa and Wilson, 2016)). ANLN is thereby proposed to serve as a prognostic marker for breast cancer. The specific function of ANLN in TNBC and the molecular mechanism by which ANLN is being regulated, however, is elusive. Our recent study provided direct evidence that TNBC-specific super-enhancer drives ANLN expression, and promotes TNBC clonogenicity (Huang et al., 2021), a sensitive indicator of undifferentiated CSCs. Owing to their clinical implications in metastasis, drug resistance and aggressiveness of tumor (Korkaya et al., 2012; Batlle and Clevers, 2017), it is important to dissect mechanisms of CSC maintenance. In the present study, we extend our effort to investigate the role of ANLN in TNBC spheroid growth and stemness. Using Crispr/Cas9 genomic editing, we delete ANLN and find a significant reduction of spheroid and clonogenic growth of TNBC, as well as impairment of mammosphere formation. Our findings further reveal the critical function of TWIST1 and BMP2 in mediating ANLN’s effect on TNBC stemness.

## Materials and Methods

### Cell Culture

HEK293T, BT-549 and Hs578T cells were obtained from ATCC. BT549 was maintained in RPMI 1640 medium (Gibco) supplemented with 10% FBS. HEK293T cells were cultured in Dulbecco’s modified Eagle medium (DMEM; Gibco) supplemented with 10% FBS. Hs578t cells were maintained in DMEM supplemented with 10% FBS and 10 μg/ml insulin. All cell lines obtained from cell banks listed above are tested for authentication using short tandem repeat profiling and passaged for fewer than 6 months, and routinely assayed for mycoplasma contamination.

### Antibodies

Anti-ANLN (#AMAB90660) and anti-actin (#3700) antibodies were obtained from Cell Signaling Technology. Anti-HA tag antibody (#11867423001) was obtained from Roche. Anti-TWIST1 (#A3237), and anti-BMP2 (#A0231) antibodies were purchased from ABclonal. Horse peroxidase-conjugated anti-mouse and anti-rabbit immunoglobulin G (IgG) antibodies (AP307P, AP308P) were obtained from Millipore. Horse peroxidase-conjugated anti-rat IgG (#AS028) was obtained from ABclonal.

### Plasmids

To knockout ANLN, Crispr/Cas9 inducible knockout system was used. FUCas9Cherry (#70182) and FgH1tUTG (#70183) were ordered from Addgene. Guide RNAs (gRNAs) were designed using online tools http://crisper.mit.edu/ and http://crisprscan.org. gRNAs oligos (Supplementary Table S1) with sticky end were synthesized by IDT company. The gRNAs were cloned into restriction BsmBI restriction sites of FgH1tUTG vector. For overexpression of exogenous BMP2-HA, CDS of BMP2 with HA tag at C-terminal was synthesized and cloned into vector CD532A-1 by GENEWIZ. To overexpress HA-TWIST1, CDS of TWIST1 with HA-tag at N-terminal were synthesized and cloned into vector CD532A-1 by GENEWIZ. For peak deletion of super-enhancer, a pair of gRNAs flanking the peak were designed using http://crisper.mit.edu/ (Supplementary Table S1). The pair of gRNAs were then inserted sequentially into BsaI and BbsI restriction sites of pX333 vector (Addgene #64073) that encodes spCas9 and two gRNA cassettes.

### Lentivirus Infection

To prepare lentiviral supernatants, 6.3 µg (FgH1tUTG and FUCas9Cherry), 7.6 µg (HA-TWIST1) or 8.2 µg (HA-BMP2) of lentiviral vectors were co-transfected with 7 µg of psPAX2 and 2.4 µg of VSV-G vectors to HEK293T cells using polyethylenimine as transfection reagent. The lentiviruses were filtered and collected after 72 h of transfection, using 0.45 µm syringe filter (Thermo fisher 7232545). 0.5 ml of lentivirus with 2 μg/ml polybrene were added to breast tumor cells for 12–24 h in a well of 6-well plates. Cells were sorted by Florescent-activated cell sorting (FACS) with cell sorter (Sony) or selected with puromycin for 5–7 days.

### Immunoblotting

Cells were washed with PBS at 4°C and lyzed in EBC buffer (0.5 NP-40, 120 mM NaCl, 50 mM Tris-HCl (pH 7.4), proteinase inhibitor cocktail, 50 mM calyculin, 1 mM sodium pyrophosphate, 20 mM sodium fluoride, 2 mM EDTA, 2 mM EGTA) for 25 min on ice. Cell extracts were pre-cleared by centrifugation at 13,000 × g for 10 min at 4°C and protein concentration was measured with Bio-Rad protein assay reagent using BioTek Synergy ™ H1 Microplate Reader. Lysates were then resolved on 10% acrylamide gels by SDS-PAGE and transferred electrophoretically to nitrocellulose membrane (Bio-Rad) at 160 mA for 80 min. The blots were blocked in TBST buffer (10 mM Tris-HCl, pH 8, 150 mM NaCl, 0.2% Tween 20) containing (w/v) non-fat dry milk 30 min, and then incubated with the specific primary antibody diluted in blocking buffer at 4°C overnight. Membranes were washed three times in TBST and incubated with horseradish peroxidase-conjugated secondary antibody for 1 h at room temperature. Membranes were washed 3 times and developed using enhanced chemiluminescence substrate (Pierce).

### Clonogenic Growth Assays

Cells were seeded to 6-well plate at a density of 800 cells/well and cultured for 10 days. Medium was changed every 4 days. After 10 days, cells were fixed with 4% formaldehyde for 15 min at room temperature. 0.1% crystal violet was then used to stain colonies for 40 min followed by washing with PBS. Images were captured and the colony number was counted.

### CellTiter-Glo® 3D and 2D Cell Viability Assays

3D cultures were prepared as previously described (Debnath et al., 2003). Briefly, 96-well plates (Corning #3610) were coated with growth factor-reduced Matrigel (BD Biosciences) and allowed to solidify for 30 min. The cells were seeded in assay medium on Matrigel-coated 96-well plate, with cell density of 2,500–4,000 cells per well. Assay medium contained DMEM/RPMI-1640 supplemented with 10% Tetracycline Free FBS and 2% Matrigel for BT549. Assay medium for Hs578T contained DMEM supplemented with 10% Tetracycline Free FBS, insulin (10 μg/ml) and 2% Matrigel. Cells coated with Matrigel were then allowed to interact with extracellular matrix and grow in 5% CO_2_ humidified incubator at 37°C to form spheroids in 7–11 days. The assay medium was replaced every 4 days. Cells were treated with 100 ng/ml doxycycline every 2 days to induce ANLN knockdown. To quantify spheroid growth and viability, CellTiter-Glo® 3D Cell Viability Assay (Promega #G9682) was performed by following instructions on product manual. To assess cell viability in 2D culture, CellTiter- Glo® Luminescent Cell Viability Assay (Promega #G7571) was used. 3,000 cells per well were seeded to 96-well plate (Corning #3610) and cultured for 3 days 50 µL reagent was then added to each well and incubated on a shaker for 15 min. Signal was read by a Synergy™ H1 Microplate Reader (BioTek).

### Mammosphere Formation Assay

Cells were seeded to ultra-low attachment 6-well plates (Corning 3,471) with cell density of 2,500 and 3,000 cells per well for BT549 and HS578t, respectively. Cells were cultured in mammosphere medium, containing DMEM/F12 supplemented with B27 (Gibco 12587010) and 20 ng/ml EGF (R&D 236-EG), for 7–8 days. Images of mammospheres were captured by Nikon NIS-Elements D software. Mammosphere with diameter >50 µm were counted using the Nikon NIS-Elements D software.

### RT-qPCR

Mammosphere samples were cultured with mammosphere medium in ultra-low attachment 10-cm plates for 7 days. Total RNA from mammosphere cultures was extracted using RNeasy Plus Mini Kit (Qiagen #74134) following the manufacturer’s instructions. Reverse transcription was performed using TaqMan Reverse transcription Reagents (Applied Biosystems, N8080234). Quantitative RT-PCR was performed using QuantStudio 12K Flex Real-Time PCR System (Applied Biosystems).

## Results

### Depletion of Anilin Inhibits Triple-Negative Breast Cancer Spheroid Growth

**FIGURE 1:**
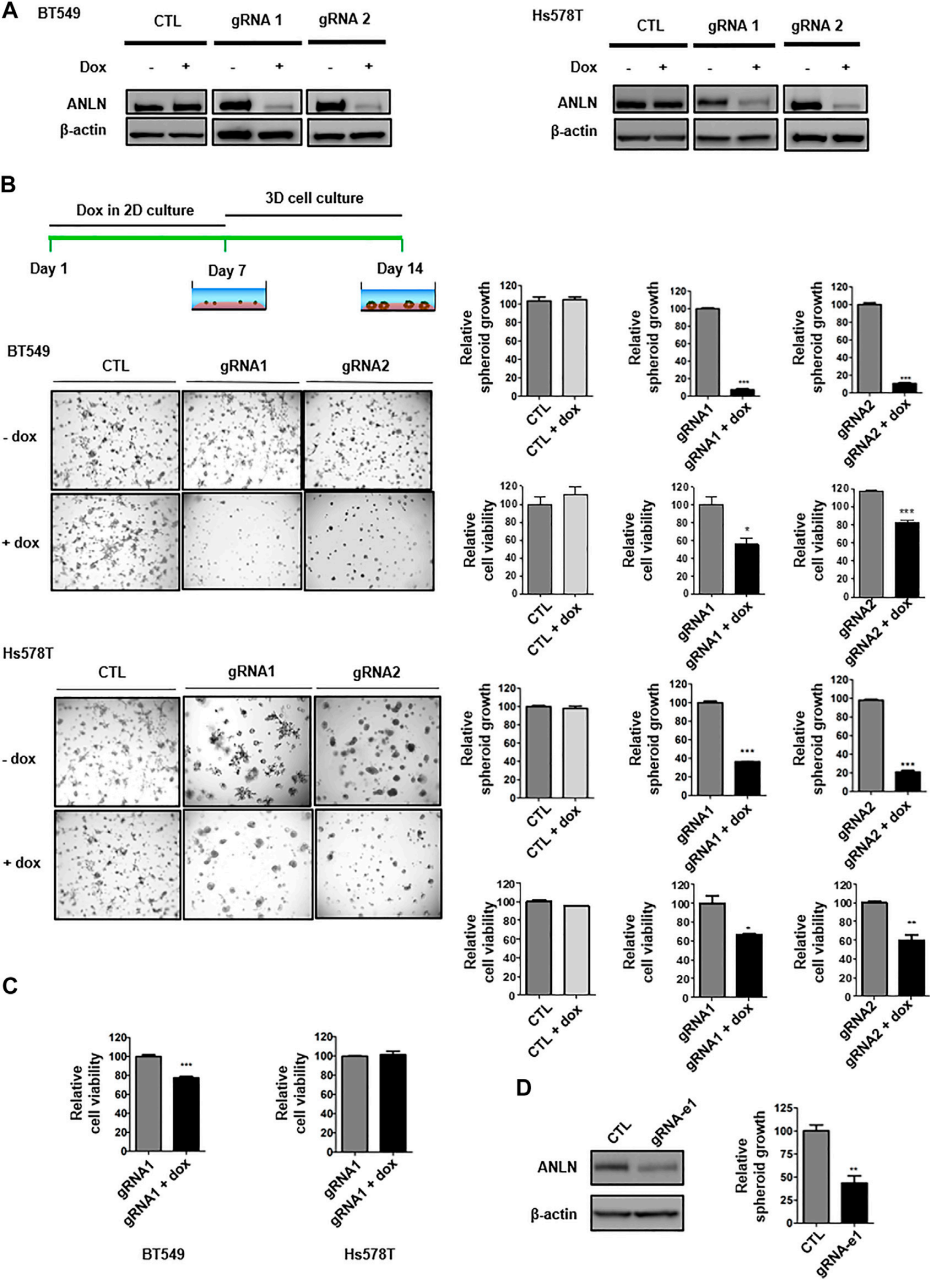
ANLN depletion attenuates TNBC spheroid growth. **(A)** BT549 and Hs578T cells expressing tet-on ANLN gRNA or vector control (CTL) were treated with doxycycline (Dox; 100 ng/ml) for 7 days. Whole-cell lysates were subjected to immunoblotting. Experiments were repeated at least 3 times with similar results. **(B)** Schematics of dox treatment and 3D culture. BT549 and Hs578T cells were infected with tet-on ANLN or CTL gRNA. Cells were cultured in 3D for 7–9 days, and images were captured. Area of spheroids was measured using NIS-Elements D software. Bar graphs depict growth of spheroids with or without ANLN knockout. Error bars, mean ± SEM of 3 independent experiments. Cell viability was assessed by 3D cell titer-Glo assay, and depicted in the bar graphs (*n* = 3). Cell viability results are representative of 3 independent experiments. *, *p* < 0.05; **, *p* < 0.01; ***, *p* < 0.001. **(C)** BT549 and Hs578T cells were infected with tet-on ANLN or CTL gRNA. Cells were cultured in 2D for 3–5 days, followed by cell titer-Glo Luminescent cell viability assay; Bar graphs depict growth of BT549 and Hs578T cells with or without ANLN knockout. Error bars, mean ± SEM of 3 independent experiments. ***, *p* < 0.001. **(D)** Immunoblotting detection of ANLN in BT549 upon deletion of e1 super-enhancer of SSE256, experiment was repeated twice with similar results. Bar graph depicts growth of spheroid with and without deletion of e1 in BT549 cells. Error bars, mean ± SEM of 3 independent experiments. **, *p* < 0.01.

To explore the function of ANLN in growth and stemness of TNBC, we generated a panel of breast cancer lines (BT549 and Hs578T) with tet-on doxycycline (dox)-inducible Crispr/Cas9-mediated knockout of ANLN. TNBC cells were infected with lentiviral vectors, followed by florescent-activated cell sorting for double-positive cells containing both GFP (dox-inducible gRNA) and mCherry (spCas9). Upon dox administration, ANLN was depleted significantly with two distinct gRNAs (Figure 1A). We then investigated the consequence of ANLN knockout on TNBC cell proliferation. The 3D spheroid morphogenesis assay, that more accurately recapitulates phenotypes governing tumor growth *in vivo,* was performed. As shown in the representative images and bar graphs, depletion of ANLN in TNBC lines inhibited spheroid growth significantly in 3D cultures (BT549: gRNA1 93% inhibition, gRNA2 91% inhibition; Hs578T: gRNA1 63% inhibition, gRNA2 79% inhibition). Conversely, dox administration in vector-control cells had no effect (Figure 1B). We have also quantified viability of cells in 3D spheroids, and shown that knockout of ANLN resulted in significant reduction of cell viability (Figure 1B). Interestingly, ANLN depletion only led to 20% inhibition of BT549 cell viability in 2D, and it had no effect on Hs578T cells in 2D cultures (Figure 1C). As our recent paper demonstrated the regulation of ANLN expression by super-enhancer in TNBC, we examined the effect of super-enhancer on spheroid growth. Deletion of ANLN-associated super-enhancer by Crispr/Cas9 editing resulted in reduction of ANLN protein expression as well as impairment of spheroid growth (Figure 1D), agreeing with the functional significance of super-enhancer in promoting ANLN expression and its associated tumorigenic phenotype.

### Knockout of Anilin Reduces Stemness of Triple-Negative Breast Cancer Cells

**FIGURE 2:**
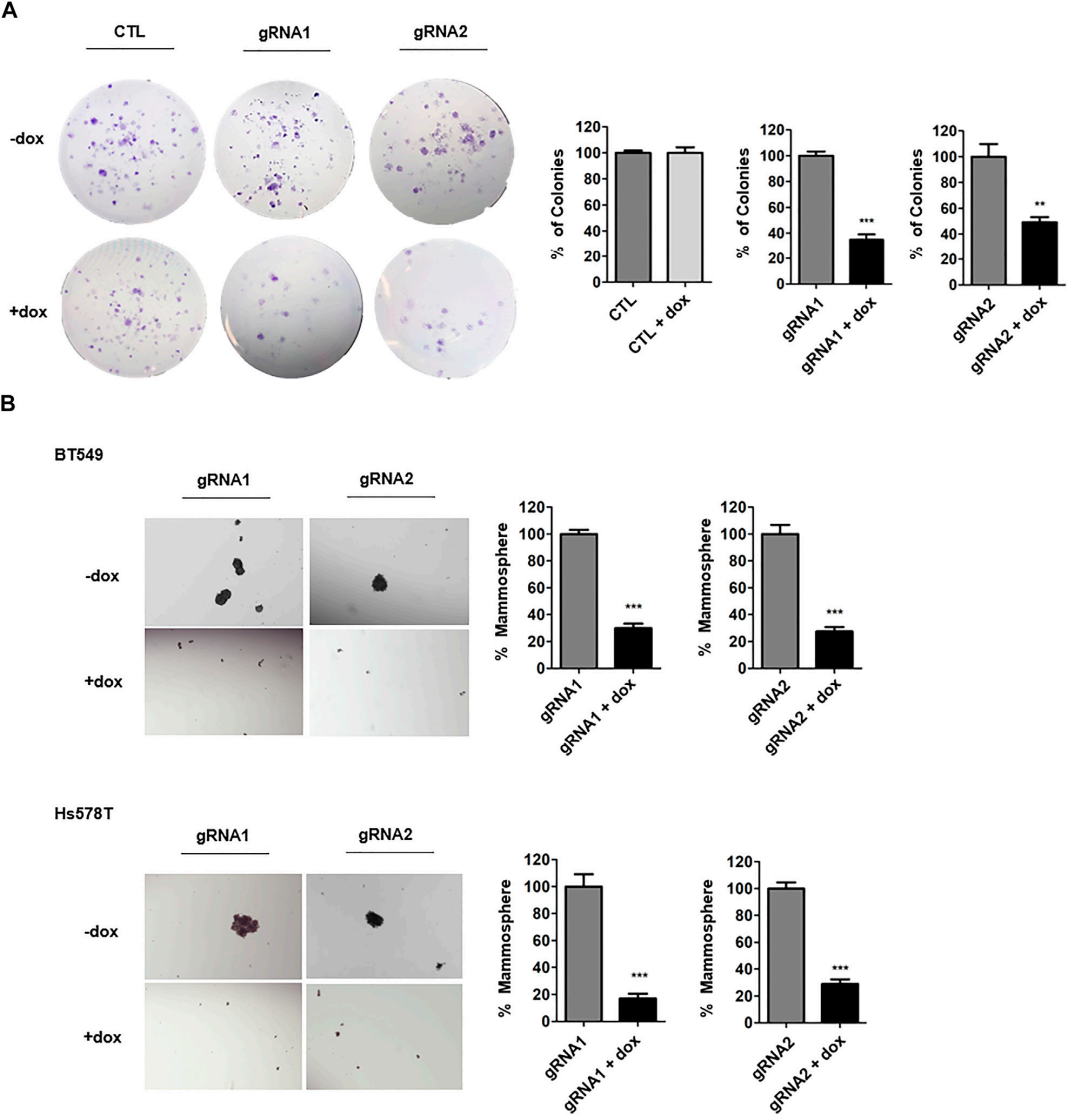
ANLN promotes stem cell property of TNBC. **(A)** BT549 cells expressing tet-on ANLN or CTL gRNA were treated with dox (100 ng/ml) for 7 days. Cells were then cultured for colony formation assay for 10 days. Representative images are shown. Colony number was counted and depicted in bar graphs. Error bars, mean ± SEM of 3 independent experiments. ***, *p* < 0.001. **(B)** BT549 and Hs578T cells expressing tet-on ANLN or CTL gRNA were treated with dox (100 ng/ml) for 7 days. Cells were then seeded for mammosphere formation assay. Representative images are shown. Bar graphs depict the mammosphere number. Error bars, mean ± SEM of 3 independent experiments. ***, *p* < 0.001.

To examine progeny producing capability, clonogenic assays were performed. The ANLN-knockout TNBC cells were seeded at low density and then cells were allowed to grow for 10 days. Using crystal violet dye, cells were stained. We showed that ANLN knockout greatly reduced the colony formation abilities of BT549 and Hs578T cells (Figure 2A, Supplementary Figure S1). In cells containing control gRNA, dox treatment had no effect on colony numbers. Next, we examined the functional contribution of ANLN on stemness of TNBC cells, by performing mammosphere formation assay. In mammosphere assay, only anoikis-resistant CSCs survive in suspension in the specific culture medium. The number of spheres present in culture reflects the number of cells which are capable of forming new tumor spheroids (Soule and McGrath, 1986; Dontu et al., 2003). TNBC cells were treated with dox for 7 days to induce ANLN knockout and then grown in mammosphere culture medium for 7–9 days. Spheres larger than 50 µm were considered as mammospheres. Using two independent gRNAs, we showed that ANLN-depleted samples (dox treated) had significantly lower number of mammospheres (Figure 2B; BT549: gRNA1 70% inhibition, gRNA2 72% inhibition; Hs578T: gRNA1 83% inhibition, gRNA2 71% inhibition). These data suggest that depletion of ANLN results in suppressed CSC properties of TNBC cells.

### Anilin Promotes Mammosphere Formation via TWIST1 and BMP2

**FIGURE 3:**
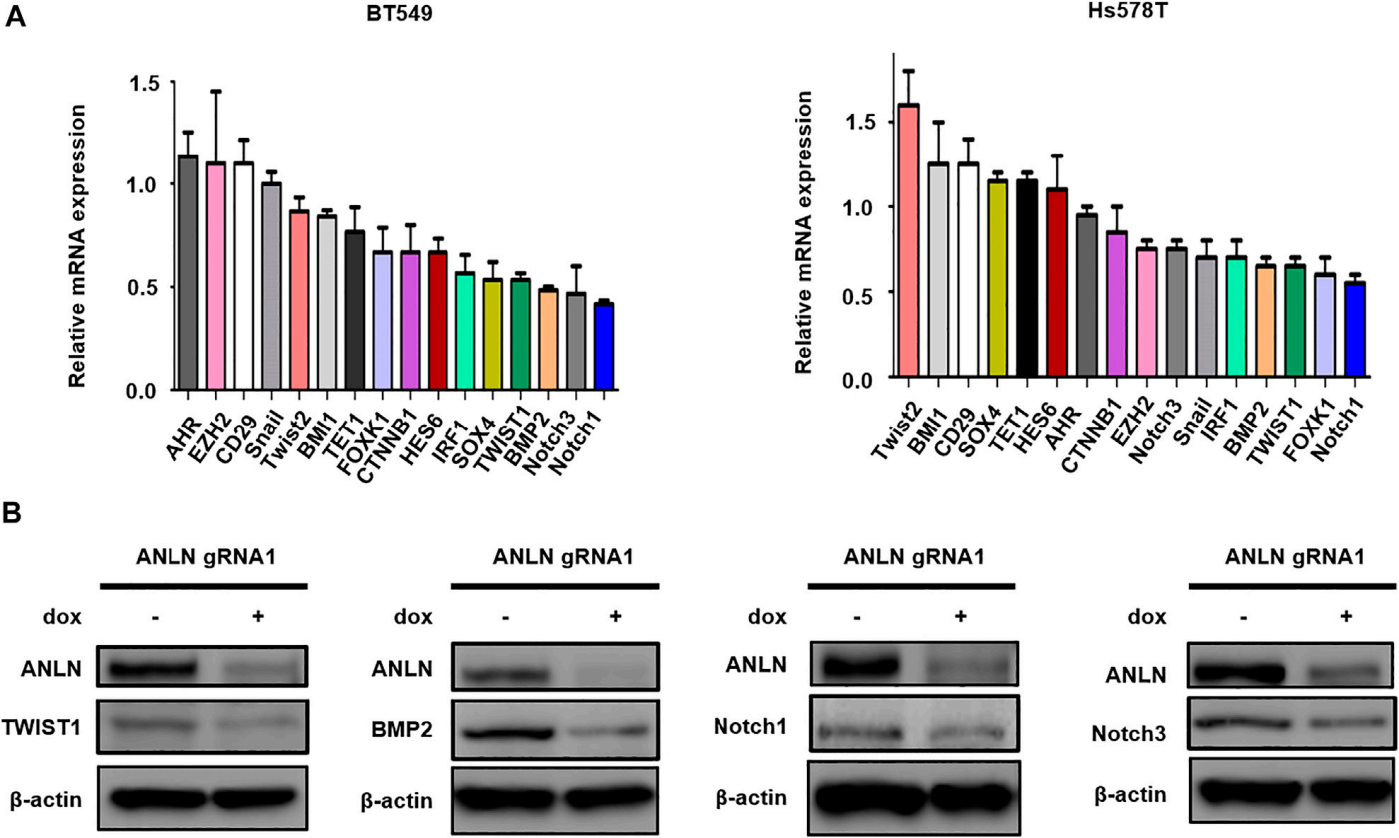
Effect of ANLN on the expression of stemness-related genes. **(A)** Bar graphs depicting mRNA expression of stemness-related genes in dox-treated (ANLN-knockout) mammospheres compared to mammospheres without dox treatment. Error bars, mean ± SEM of 3 independent experiments. **(B)** Immunoblot showing expression level of Twsit1, Bmp2, Notch1 and Notch3 proteins in Hs578T cells with and without ANLN knockout. Experiments were repeated twice independently with similar results.

**FIGURE 4:**
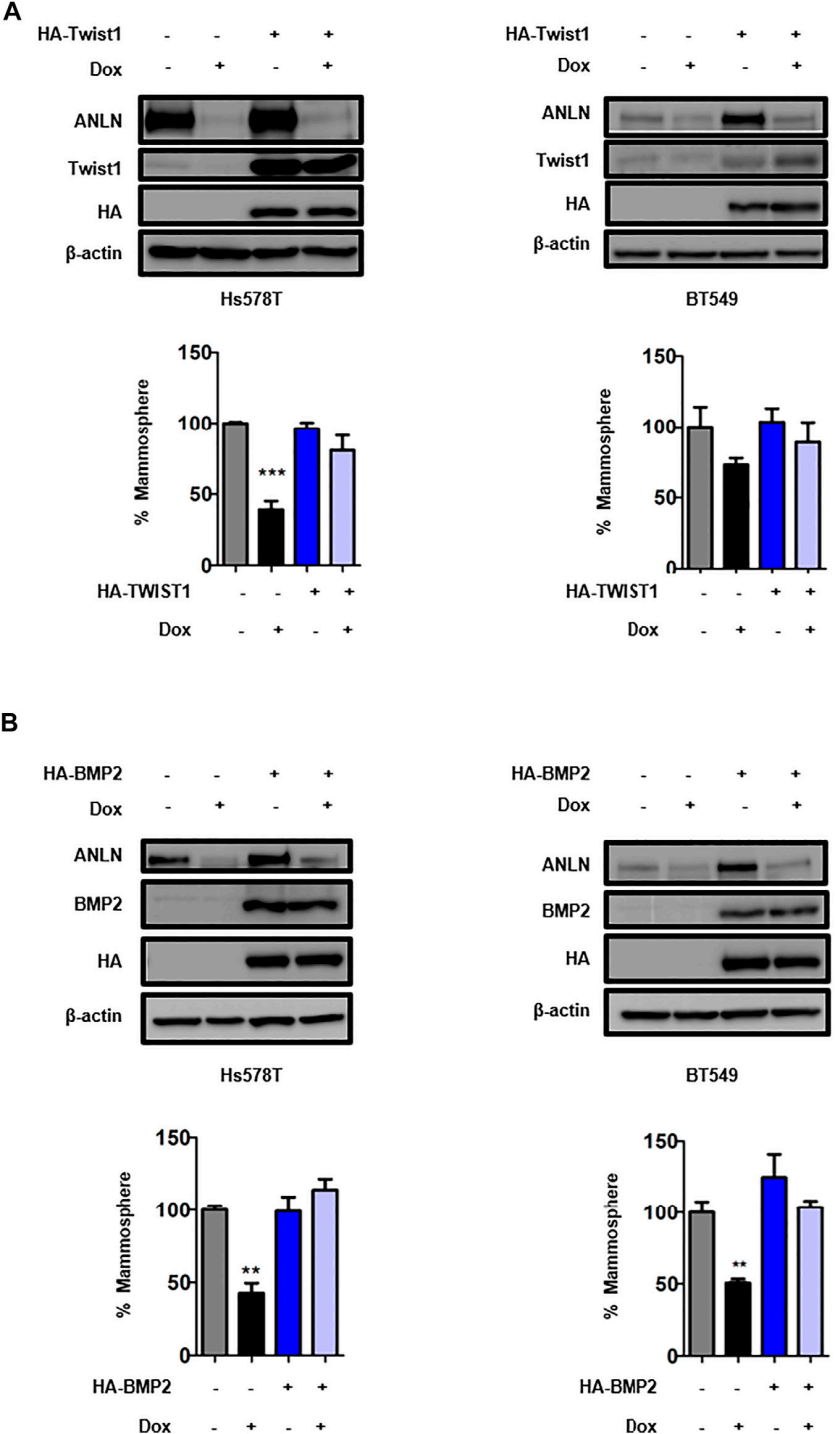
TWIST1 and BMP2 are critical downstream effectors of ANLN in regulating TNBC CSCs. **(A)** Hs578t and BT549 cells expressing HA-Twist1 or control vector were infected with tet-on ANLN gRNA1. Cells were treated with dox (100 ng/ml) for 7 days, and then subjected to mammosphere formation assay. Error bars, mean ± SEM of 3 independent experiments. Whole cell lysates were subjected to Immunoblotting analysis. ***, *p* < 0.001. **(B)** Hs578t and BT549 cells expressing HA-Bmp2 or control vector were infected with tet-on ANLN gRNA1. Cells were treated with dox (100 ng/ml) for 7 days, and then subjected to mammosphere formation assay. Error bars, mean ± SEM of 3 independent experiments. Whole cell lysates were subjected to Immunoblotting analysis. **, *p* < 0.01.

To dissect the mechanism by which ANLN regulates stemness in TNBC cells, a panel of stem cell-related genes were screened using RT-qPCR. Figure 3A and Supplementary Table S2 show the mRNA levels of CSC-related genes in ANLN-depleted mammospheres. Among these, 4 of them (TWIST1, BMP2, Notch1, Notch3) were inhibited to the greatest extent in ANLN-depleted BT549 mammospheres. Decreased expression of these 4 proteins in ANLN-knockout cells was also confirmed by immunoblot analysis (Figure 3B). As TWIST1 and BMP2 have been shown to promote CSC properties in breast cancer (Vesuna et al., 2009; Li and Zhou, 2011; Huang et al., 2017), we overexpressed these two genes to determine the role of them in mediating ANLN’s function. The overexpression of these proteins was confirmed by immunoblot analysis in TNBC cells (Figure 4). Next, we investigated the effect of TWIST1 on the regulation of CSC properties using mammosphere formation assay. Overexpression of HA-TWIST1 rescued the mammosphere formation ability in ANLN-depleted BT549 and Hs578T cells (Figure 4A). Similar results were observed for HA-BMP2 overexpression (Figure 4B). However, overexpression of TWIST1 or BMP2 did not rescue spheroid growth of ANLN-depleted cells (Supplementary Figure S2). These data indicated that TWIST1 and BMP2 mediate, at least in part, the function of ANLN in promoting stemness of TNBC cells.

## Discussion

By leveraging the TNBC-specific epigenomic data, we recently identified ANLN as a TNBC-specific gene regulated by super-enhancer (Huang et al., 2021). In the present study, we examined the functional role of ANLN in TNBC by knocking out the gene using Crispr/Cas9 approach. We first examined the effect of ANLN depletion on spheroid growth in 3D cultures. Our 3D system contains Matrigel, which is enriched in basement membrane components including laminin, collagen and entactin. These components resemble closely to the complex extracellular environment present in breast tumors (Badea et al., 2019), and our results demonstrated a potent impairment of spheroid growth upon ANLN depletion. Interestingly, when these ANLN-depleted cells were grown in 2D cultures, the effect of ANLN knockout on cell proliferation was minimal. Our data agree with a recent study on breast cancer, where neither ANLN knockdown nor overexpression affected cell proliferation of breast cancer cells grown in 2D *in vitro* (Wang et al., 2020). Similarly, previous studies by us and others have reported differences in proliferation and metabolic capability between 3D and 2D cultured cells. In prostate cancer, whereas silencing Akt2 in spheroids and xenografts induces robust apoptosis, knocking down Akt2 in 2D culture has minimal effect on cell survival (Chin et al., 2014). In colon cancer, 3D spheroids display low activities of mTOR, S6K and Akt signaling pathways compared to cells grown in 2D cultures (Riedl et al., 2017), suggesting distinct signaling rewiring in 3D environment. Since ANLN interacts with RhoA, which function has been shown to be modulated directly by interaction with the extracellular matrix (Lim et al., 2010), it is possible that the potent effect of ANLN on TNBC cell proliferation in 3D cultures is contributed by the interaction of extracellular matrix proteins with intracellular actin machinery and ANLN. In recent years, there have been advances in the development of 3D culture systems for mechanistic studies as well as drug screening. Commonly used models include scaffold-free, scaffold-based and hybrid 3D systems, each has their own merits and limitations (Langhans, 2018). Scaffold-free model, relying on self-aggregation of cells, allows formation of more uniform spheroids but not readily mimics cell-extracellular matrix interactions. On the other hand, scaffold-based system with Matrigel provides a good mimic of *in vivo* matrix environment yet may have the issue of batch-to-batch variability. Our laboratory routinely uses the Matrigel overlay method (cells embedded in Matrigel) as well as method involved seeding cells in medium/2% Matrigel to plates pre-treated with ultra-low attachment coating, and find no difference in spheroid morphology between the two methods (Chin et al., 2014). It would be interesting to perform co-culture 3D experiments in microplates which would allow assessment of ANLN function in a complex tumor microenvironment.

The role of ANLN in cancer stemness has not been well-studied. Nevertheless, ANLN is implicated in self-renewal of progenitor cells in developing zebrafish. A study reported that during asymmetric cell division of retinal ganglion cells, ANLN plays a critical role in progenitor cell self-renewal and balances the asymmetric and symmetric outcomes that are important for correct neurogenesis in the retina (Paolini et al., 2015). Recently, Wang et. al. has shown an important role of ANLN in promoting breast cancer stemness (Wang et al., 2020). The authors further performed RNAseq analysis in ANLN-depleted cells and showed that a number of known regulators of stemness and differentiation such as OVOL2, TBX18, FOXK1, SOX-9 and PBX1 were downregulated. The authors proposed that these genes may be involved in ANLN-mediated stemness, but there is lack of experimental support for the connections. We, therefore, first investigated the role of ANLN in CSC regulation in TNBC by performing mammosphere formation assay. Significant decrease in mammosphere numbers was observed in ANLN-depleted cells, indicating a prominent function of ANLN in CSC regulation. These results prompted us to explore the unknown mechanism of CSC regulation by ANLN. Using RT-qPCR, we identified several stem cell-related genes including TWIST1 and BMP2 that were suppressed in ANLN-depleted cells. In rescue experiments, we further demonstrated that TWIST1 and BMP2 successfully restored the mammosphere formation ability of ANLN-depleted TNBC cells. Interestingly, TWIST1 and BMP2 did not rescue spheroid growth in ANLN-depleted cells. As mammosphere assay medium enriches CSC survival and growth, whereas 3D culture with Matrigel allows TNBC cells grow and proliferate by interacting with extracellular matrix, our data support the role of TWIST1 and BMP2 as effectors of ANLN for promoting CSC properties that have not been reported previously. In addition to TWIST1 and BMP2, our finding suggests that ANLN regulates expression of other stemness-related genes including Notch1 and Notch3. Whether these genes mediate ANLN’s function in CSCs and tumorigenesis await further investigation.

TWIST1 is a transcription factor that plays an important role in driving the process of epithelial mesenchymal transition (EMT) during development and in cancer (Yang et al., 2004). Various studies have also demonstrated the role of TWIST1 in cancer stemness. Overexpression of TWIST1 in breast cancer cell lines were shown to promote the ability of mammosphere to self-renew *in vitro* as well as promote tumor initiation ability in immunodeficient mice (Mani et al., 2008; Morel et al., 2008). Another study showed that transient activation of TWIST1 is responsible for promoting stemness without induction of EMT (Jung and Yang, 2015). Upstream of TWIST1, Metadherin (MTDH) was shown to epigenetically activate TWIST1 to promote stem-like traits in breast cancer (Liang et al., 2015). MTDH is transmembrane protein that contributes to the growth, metastasis, drug resistance and relapse of tumor. It facilitates histone 3 acetylation of the TWIST1 promotor, by interacting with and preventing degradation of histone acetyltransferase CBP. Interestingly, MTDH silencing in gastric cancer cells regulates actin cytoskeletal remodeling (Du et al., 2017). Given the link between ANLN and actin cytoskeleton, it is possible that ANLN interacts with MTDH, which in turn promotes the transcription of TWIST1 in TNBC.

Bone morphogenetic proteins (BMPs) belong to the superfamily of transformation growth factor TGF-β. They were originally identified for their function in osteogenesis and bone turnover. Since then, extensive studies have investigated their role in various cancers (Hogan, 1996; Hou et al., 2009; Liao et al., 2015). BMP signaling has been demonstrated to promote or inhibit cancer growth and progression in different tumor contexts. In renal carcinoma and osteosarcoma, BMP2 inhibits cancer stemness (Wang et al., 2011; Wang et al., 2015). In contrast, the positive role of BMP2 in CSC regulation was observed in glioblastoma and colon cancer (Piccirillo and Vescovi, 2007; Kim et al., 2015). Our study indicates that BMP2 promotes TNBC stemness, which is concordant with another study which shows a critical function of BMP2 in promoting breast cancer EMT and stemness through Rb signaling pathway (Huang et al., 2017). The molecular mechanism by which BMP2 regulates CSCs in TNBC remains to be determined. In colon cancer, knockdown of STAT3 reversed BMP2-induced CSC formation (Kim et al., 2015). Results from our qPCR experiments show downregulation of STAT3 in ANLN-depleted TNBC cells. It will be interesting to test if ANLN regulates TNBC CSCs via BMP2-STAT3 signaling axis in future studies. Taken together, the present study identified an important function of ANLN in promoting spheroid growth of TNBC. In addition, we established an integrated mechanism by which ANLN induces TNBC stemness via TWIST1 and BMP2. These findings may be helpful for developing therapeutic strategies against TNBC.

## Data Availability

The original contributions presented in the study are included in the article/Supplementary Files, further inquiries can be directed to the corresponding author.

## References

[B1] Badea M. A. Balas M. Hermenean A. Ciceu A. Herman H. Ionita D. (2019). Influence of Matrigel on Single- and Multiple-Spheroid Cultures in Breast Cancer Research. SLAS DISCOVERY: Advancing Sci. Drug Discov. 24 (5), 563–578. 10.1177/2472555219834698 30897015

[B2] Batlle E. Clevers H. (2017). Cancer Stem Cells Revisited. Nat. Med. 23 (10), 1124–1134. 10.1038/nm.4409 28985214

[B3] Cancer Genome Atlas N. (2012). Comprehensive Molecular Portraits of Human Breast Tumours. Nature 490 (7418), 61–70. 10.1038/nature11412 23000897 PMC3465532

[B4] Chin Y. R. Yuan X. Balk S. P. Toker A. (2014). PTEN-deficient Tumors Depend on AKT2 for Maintenance and Survival. Cancer Discov. 4 (8), 942–955. 10.1158/2159-8290.Cd-13-0873 24838891 PMC4125464

[B5] Debnath J. Muthuswamy S. K. Brugge J. S. (2003). Morphogenesis and Oncogenesis of MCF-10A Mammary Epithelial Acini Grown in Three-Dimensional Basement Membrane Cultures. Methods 30 (3), 256–268. 10.1016/s1046-2023(03)00032-x 12798140

[B6] Dontu G. Abdallah W. M. Foley J. M. Jackson K. W. Clarke M. F. Kawamura M. J. (2003). *In Vitro* propagation and Transcriptional Profiling of Human Mammary Stem/progenitor Cells. Genes Dev. 17 (10), 1253–1270. 10.1101/gad.1061803 12756227 PMC196056

[B7] Du Y. Jiang B. Song S. Pei G. Ni X. Wu J. (2017). Metadherin Regulates Actin Cytoskeletal Remodeling and Enhances Human Gastric Cancer Metastasis via Epithelial-Mesenchymal Transition. Int. J. Oncol. 51 (1), 63–74. 10.3892/ijo.2017.4002 28534938 PMC5467779

[B8] Herranz D. Ambesi-Impiombato A. Palomero T. Schnell S. A. Belver L. Wendorff A. A. (2014). A NOTCH1-Driven MYC Enhancer Promotes T Cell Development, Transformation and Acute Lymphoblastic Leukemia. Nat. Med. 20 (10), 1130–1137. 10.1038/nm.3665 25194570 PMC4192073

[B9] Hnisz D. Abraham B. J. Lee T. I. Lau A. Saint-André V. Sigova A. A. (2013). Super-enhancers in the Control of Cell Identity and Disease. Cell 155 (4), 934–947. 10.1016/j.cell.2013.09.053 24119843 PMC3841062

[B10] Hogan B. L. (1996). Bone Morphogenetic Proteins: Multifunctional Regulators of Vertebrate Development. Genes Dev. 10 (13), 1580–1594. 10.1101/gad.10.13.1580 8682290

[B11] Hou C.-H. Hsiao Y.-C. Fong Y.-C. Tang C.-H. (2009). Bone Morphogenetic Protein-2 Enhances the Motility of Chondrosarcoma Cells via Activation of Matrix Metalloproteinase-13. Bone 44 (2), 233–242. 10.1016/j.bone.2008.09.021 19038372

[B12] Huang H. Hu J. Maryam A. Huang Q. Zhang Y. Ramakrishnan S. (2021). Defining Super-enhancer Landscape in Triple-Negative Breast Cancer by Multiomic Profiling. Nat. Commun. 12 (1), 2242. 10.1038/s41467-021-22445-0 33854062 PMC8046763

[B13] Huang P. Chen A. He W. Li Z. Zhang G. Liu Z. (2017). BMP-2 Induces EMT and Breast Cancer Stemness through Rb and CD44. Cel Death Discov. 3 (1), 1–12. 10.1038/cddiscovery.2017.39 PMC5511860 28725489

[B14] Jung H.-Y. Yang J. (2015). Unraveling the TWIST between EMT and Cancer Stemness. Cell Stem Cell 16 (1), 1–2. 10.1016/j.stem.2014.12.005 25575073

[B15] Kim B. R. Oh S. C. Lee D.-H. Kim J. L. Lee S. Y. Kang M. H. (2015). BMP-2 Induces Motility and Invasiveness by Promoting colon Cancer Stemness through STAT3 Activation. Tumor Biol. 36 (12), 9475–9486. 10.1007/s13277-015-3681-y 26124007

[B16] Korkaya H. Kim G.-i. Davis A. Malik F. Henry N. L. Ithimakin S. (2012). Activation of an IL6 Inflammatory Loop Mediates Trastuzumab Resistance in HER2+ Breast Cancer by Expanding the Cancer Stem Cell Population. Mol. Cel 47(4), 570–584. 10.1016/j.molcel.2012.06.014 PMC3432419 22819326

[B17] Langhans S. A. (2018). Three-Dimensional *In Vitro* Cell Culture Models in Drug Discovery and Drug Repositioning. Front. Pharmacol. 9, 6. 10.3389/fphar.2018.00006 29410625 PMC5787088

[B18] Li J. Zhou B. P. (2011). Activation of β-catenin and Akt Pathways by Twist Are Critical for the Maintenance of EMT Associated Cancer Stem Cell-like Characters. BMC cancer 11 (1), 1–11. 10.1186/1471-2407-11-49 21284870 PMC3040162

[B19] Liang Y. Hu J. Li J. Liu Y. Yu J. Zhuang X. (2015). Epigenetic Activation of TWIST1 by MTDH Promotes Cancer Stem-like Cell Traits in Breast Cancer. Cancer Res. 75 (17), 3672–3680. 10.1158/0008-5472.can-15-0930 26141861

[B20] Liao A. Wang W. Sun D. Jiang Y. Tian S. Li J. (2015). Bone Morphogenetic Protein 2 Mediates Epithelial-Mesenchymal Transition via AKT and ERK Signaling Pathways in Gastric Cancer. Tumor Biol. 36 (4), 2773–2778. 10.1007/s13277-014-2901-1 25448881

[B21] Lim S.-M. Kreipe B. A. Trzeciakowski J. Dangott L. Trache A. (2010). Extracellular Matrix Effect on RhoA Signaling Modulation in Vascular Smooth Muscle Cells. Exp. Cel. Res. 316 (17), 2833–2848. 10.1016/j.yexcr.2010.06.010 20599954

[B22] Magnusson K. Gremel G. Rydén L. Pontén V. Uhlén M. Dimberg A. (2016). ANLN Is a Prognostic Biomarker Independent of Ki-67 and Essential for Cell Cycle Progression in Primary Breast Cancer. BMC Cancer 16 (1), 904. 10.1186/s12885-016-2923-8 27863473 PMC5116155

[B23] Mani S. A. Guo W. Liao M.-J. Eaton E. N. Ayyanan A. Zhou A. Y. (2008). The Epithelial-Mesenchymal Transition Generates Cells with Properties of Stem Cells. Cell 133 (4), 704–715. 10.1016/j.cell.2008.03.027 18485877 PMC2728032

[B24] Morel A.-P. Lièvre M. Thomas C. Hinkal G. Ansieau S. Puisieux A. (2008). Generation of Breast Cancer Stem Cells through Epithelial-Mesenchymal Transition. PLoS One 3 (8), e2888. 10.1371/journal.pone.0002888 18682804 PMC2492808

[B25] Oegema K. Savoian M. S. Mitchison T. J. Field C. M. (2000). Functional Analysis of a Human Homologue of the Drosophila Actin Binding Protein Anillin Suggests a Role in Cytokinesis. J. Cel Biol. 150 (3), 539–552. 10.1083/jcb.150.3.539 PMC2175195 10931866

[B26] Ooi W. F. Xing M. Xu C. Yao X. Ramlee M. K. Lim M. C. (2016). Epigenomic Profiling of Primary Gastric Adenocarcinoma Reveals Super-enhancer Heterogeneity. Nat. Commun. 7, 12983. 10.1038/ncomms12983 27677335 PMC5052795

[B27] Paolini A. Duchemin A. L. Albadri S. Patzel E. Bornhorst D. González Avalos P. (2015). Asymmetric Inheritance of the Apical Domain and Self-Renewal of Retinal Ganglion Cell Progenitors Depend on Anillin Function. Development 142 (5), 832–839. 10.1242/dev.118612 25655700

[B28] Piccirillo S. G. M. Vescovi A. L. (2007). Bone Morphogenetic Proteins Regulate Tumorigenicity in Human Glioblastoma Stem Cells. Cancer Stem Cells, 5, 59–81. 10.1007/2789_2007_044 17939295

[B29] Piekny A. J. Maddox A. S. (2010). The Myriad Roles of Anillin during Cytokinesis. Semin. Cel Dev. Biol. 21 (9), 881–891. 10.1016/j.semcdb.2010.08.002 20732437

[B30] Riedl A. Schlederer M. Pudelko K. Stadler M. Walter S. Unterleuthner D. (2017). Comparison of Cancer Cells in 2D vs 3D Culture Reveals Differences in AKT-mTOR-S6k Signaling and Drug Responses. J. Cel Sci. 130 (1), 203–218. 10.1242/jcs.188102 27663511

[B31] Soule H. D. McGrath C. M. (1986). A Simplified Method for Passage and Long-Term Growth of Human Mammary Epithelial Cells. In Vitro Cel Dev Biol. 22 (1), 6–12. 10.1007/bf02623435 2418007

[B32] Sung H. Ferlay J. Siegel R. L. Laversanne M. Soerjomataram I. Jemal A. (2021). Global Cancer Statistics 2020: GLOBOCAN Estimates of Incidence and Mortality Worldwide for 36 Cancers in 185 Countries. CA A. Cancer J. Clin. 71, 209–249. 10.3322/caac.21660 33538338

[B33] Suzuki C. Daigo Y. Ishikawa N. Kato T. Hayama S. Ito T. (2005). ANLN Plays a Critical Role in Human Lung Carcinogenesis through the Activation of RHOA and by Involvement in the Phosphoinositide 3-kinase/AKT Pathway. Cancer Res. 65 (24), 11314–11325. 10.1158/0008-5472.Can-05-1507 16357138

[B34] Thapa R. Wilson G. D. (2016). The Importance of CD44 as a Stem Cell Biomarker and Therapeutic Target in Cancer. Stem Cell Int. 2016, 1–15. 10.1155/2016/2087204 PMC4856920 27200096

[B35] Vesuna F. Lisok A. Kimble B. Raman V. (2009). Twist Modulates Breast Cancer Stem Cells by Transcriptional Regulation of CD24 Expression. Neoplasia 11 (12), 1318–1328. 10.1593/neo.91084 20019840 PMC2794513

[B36] Wang D. Naydenov N. G. Dozmorov M. G. Koblinski J. E. Ivanov A. I. (2020). Anillin Regulates Breast Cancer Cell Migration, Growth, and Metastasis by Non-canonical Mechanisms Involving Control of Cell Stemness and Differentiation. Breast Cancer Res. 22 (1), 3. 10.1186/s13058-019-1241-x 31910867 PMC6947866

[B37] Wang L. Park P. La Marca F. Than K. D. Lin C.-Y. (2015). BMP-2 Inhibits Tumor-Initiating Ability in Human Renal Cancer Stem Cells and Induces Bone Formation. J. Cancer Res. Clin. Oncol. 141 (6), 1013–1024. 10.1007/s00432-014-1883-0 25431339 PMC11824135

[B38] Wang L. Park P. Zhang H. La Marca F. Claeson A. Valdivia J. (2011). BMP-2 Inhibits the Tumorigenicity of Cancer Stem Cells in Human Osteosarcoma OS99-1 Cell Line. Cancer Biol. Ther. 11 (5), 457–463. 10.4161/cbt.11.5.14372 21178508 PMC3230314

[B39] Yang J. Mani S. A. Donaher J. L. Ramaswamy S. Itzykson R. A. Come C. (2004). Twist, a Master Regulator of Morphogenesis, Plays an Essential Role in Tumor Metastasis. cell 117 (7), 927–939. 10.1016/j.cell.2004.06.006 15210113

[B40] Zhang X. Choi P. S. Francis J. M. Imielinski M. Watanabe H. Cherniack A. D. (2016). Identification of Focally Amplified Lineage-specific Super-enhancers in Human Epithelial Cancers. Nat. Genet. 48 (2), 176–182. 10.1038/ng.3470 26656844 PMC4857881

[B41] Zhou W. Wang Z. Shen N. Pi W. Jiang W. Huang J. (2015). Knockdown of ANLN by Lentivirus Inhibits Cell Growth and Migration in Human Breast Cancer. Mol. Cel Biochem. 398 (1), 11–19. 10.1007/s11010-014-2200-6 25223638

