# Supplementary material for: ANLN Enhances Triple-Negative Breast Cancer Stemness Through TWIST1 and BMP2 and Promotes its Spheroid Growth
Source: Front Mol Biosci. 2021 Jul 1;8:700973. doi: 10.3389/fmolb.2021.700973 (PMC8280772; doi:10.3389/fmolb.2021.700973)
Supplement: Supplementary file 2 [file DataSheet1.pdf]

SUPPLEMENTARY INFORMATION

ANLN ENHANCES TRIPLE-NEGATIVE BREAST CANCER STEMNESS THROUGH TWIST1 AND BMP2 AND PROMOTES ITS SPHEROID GROWTH

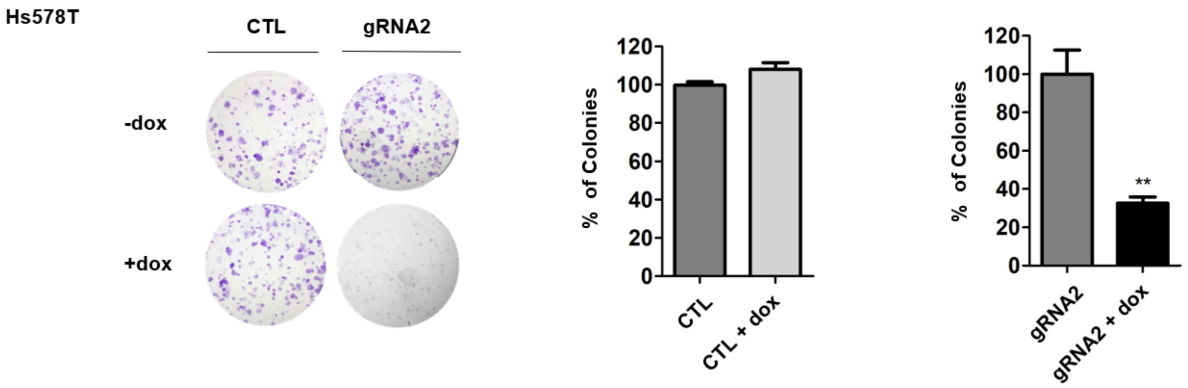

Supplementary Figure 1. ANLN depletion suppresses colony formation in Hs578T cells. Hs578T cells expressing tet-on ANLN were treated or CTL gRNA were treated with dox (100 ng/ml) for 7 days. Cells were then cultured for colony formation assay for 10 days. Representative images are shown. Colony number was counted and depicted in bar graph. Error bars, mean ± SEM of 3 independent experiments. ***, $p < 0.001$.

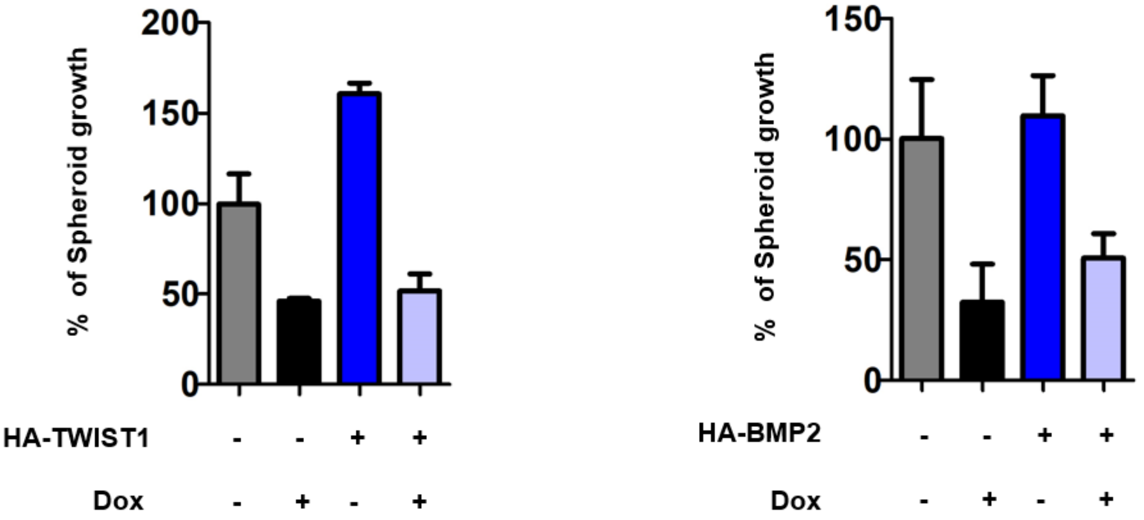

Supplementary Figure 2. TWIST1 and BMP2 do not rescue spheroid growth of ANLN-depleted cells.

BT549 cells expressing HA-twist1, HA-bmp2 or control vector were infected with tet-on ANLN gRNA1. Cells were treated with dox (100 ng/ml) for 7 days, and then cultured in 3D for 7 to 9 days followed by 3D cell titer-Glo assay. Bar graphs depict relative spheroid growth of BT549. Error bars, mean ± SEM of 2 independent experiments. *, $p < 0.05$.
